# Supplementary figures and images for: Transcriptional factor regulation network and competitive endogenous RNA (ceRNA) network determining response of esophageal squamous cell carcinomas to neoadjuvant chemoradiotherapy
Source: PeerJ. 2019 Mar 29;7:e6668. doi: 10.7717/peerj.6668 (PMC6442670; doi:10.7717/peerj.6668)

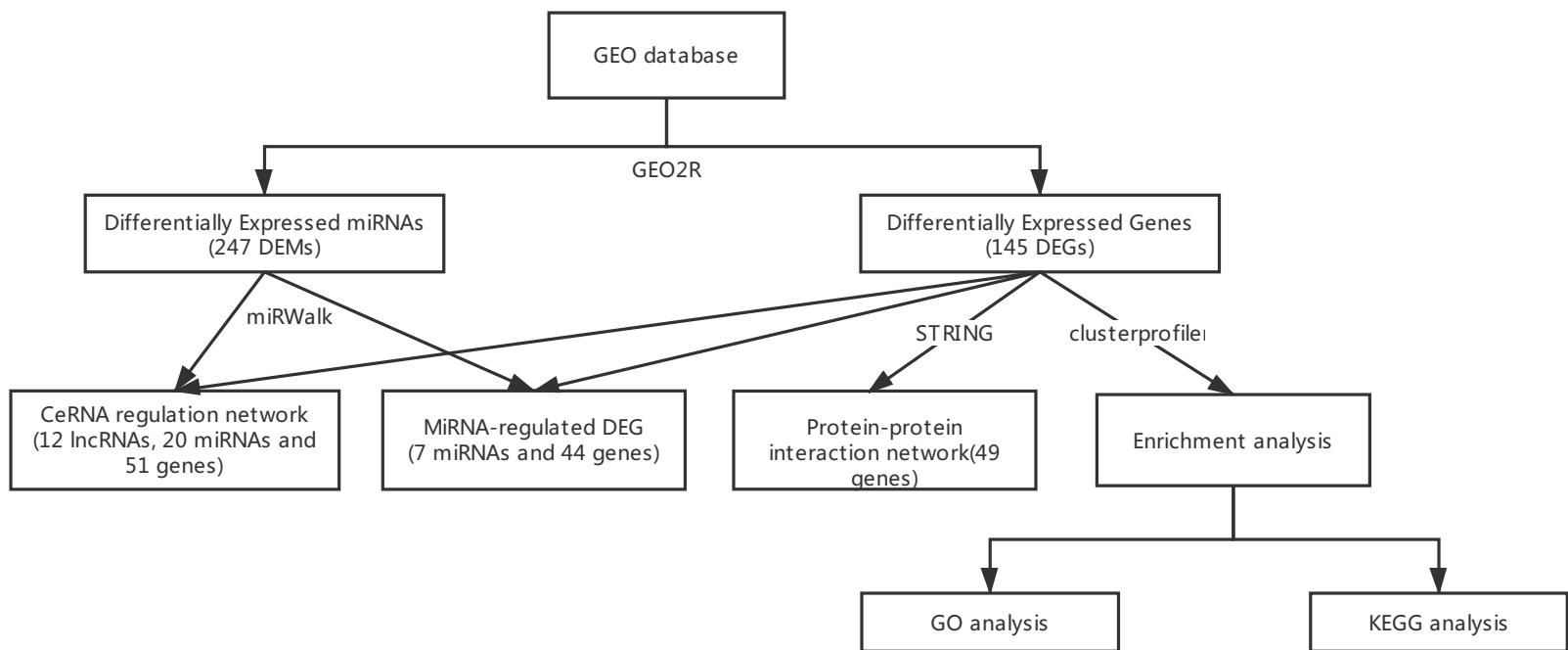

Supplement: Figure S1 [file peerj-07-6668-s001.pdf]

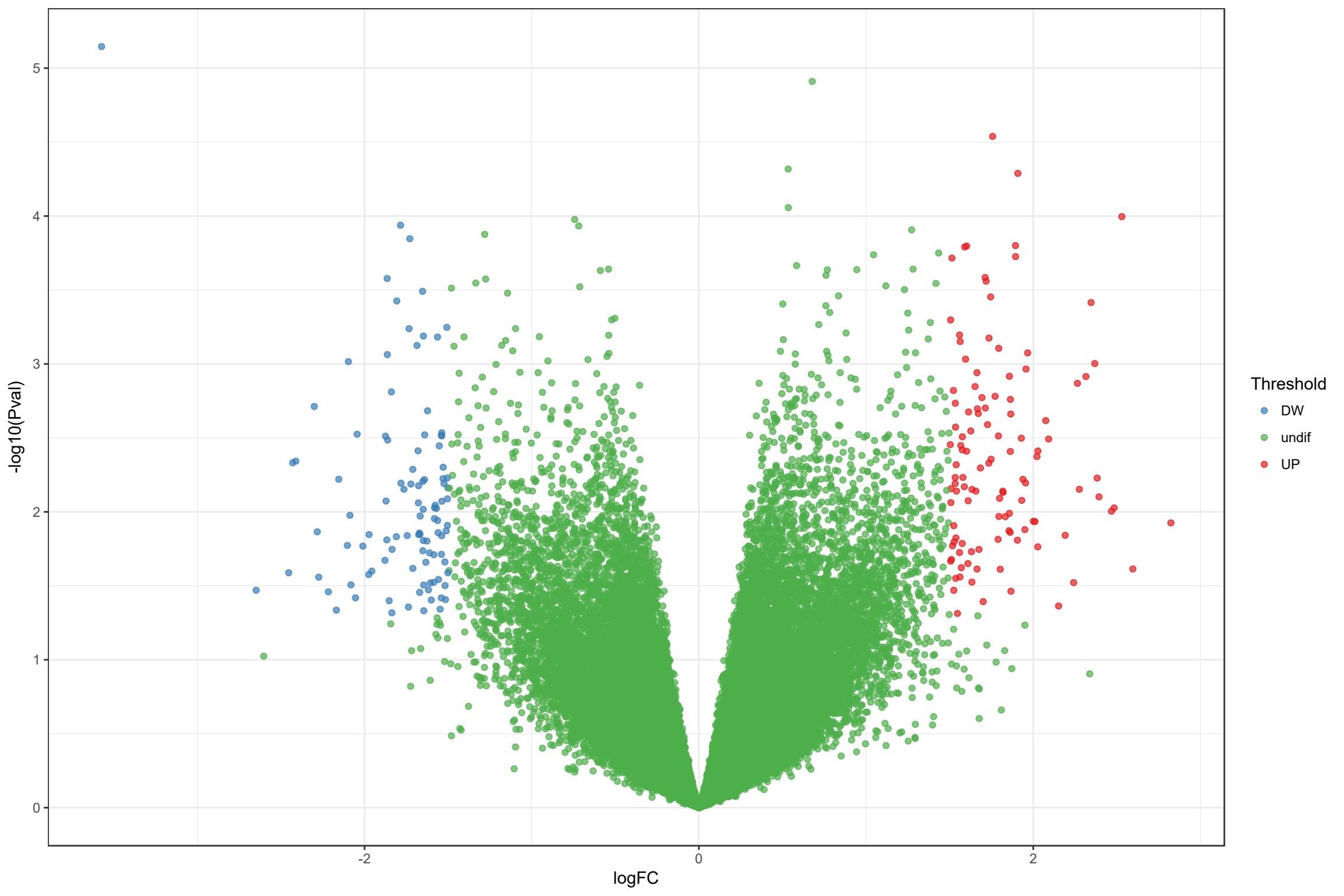

Supplement: Figure S2 — Green represents no difference, blue represents low expression in the response group, and red represents high expression in the response group. [file peerj-07-6668-s002.pdf]

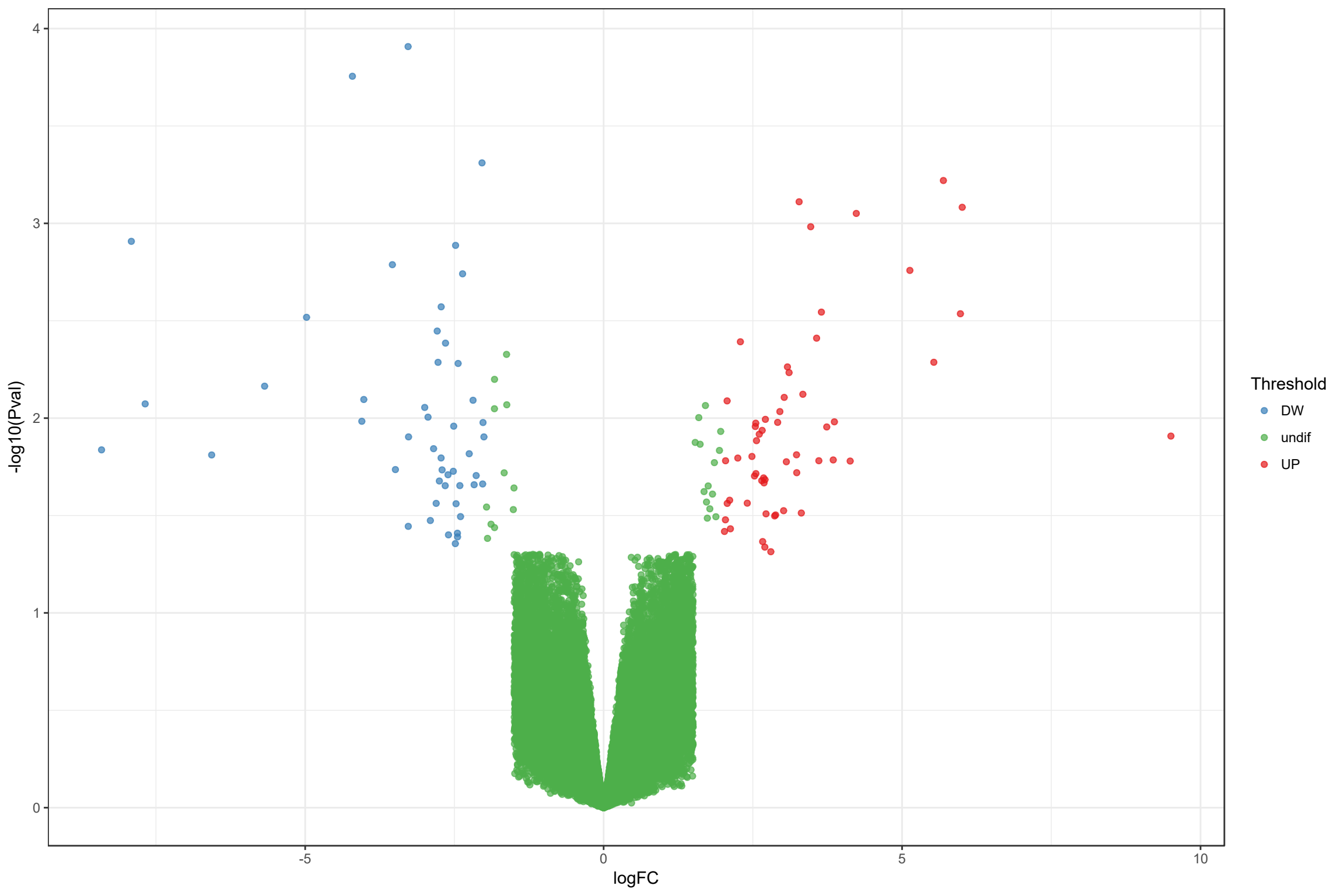

Supplement: Figure S3 — Green represents no difference, blue represents low expression in the response group, and red represents high expression in the response group. [file peerj-07-6668-s003.pdf]

A

hub gene

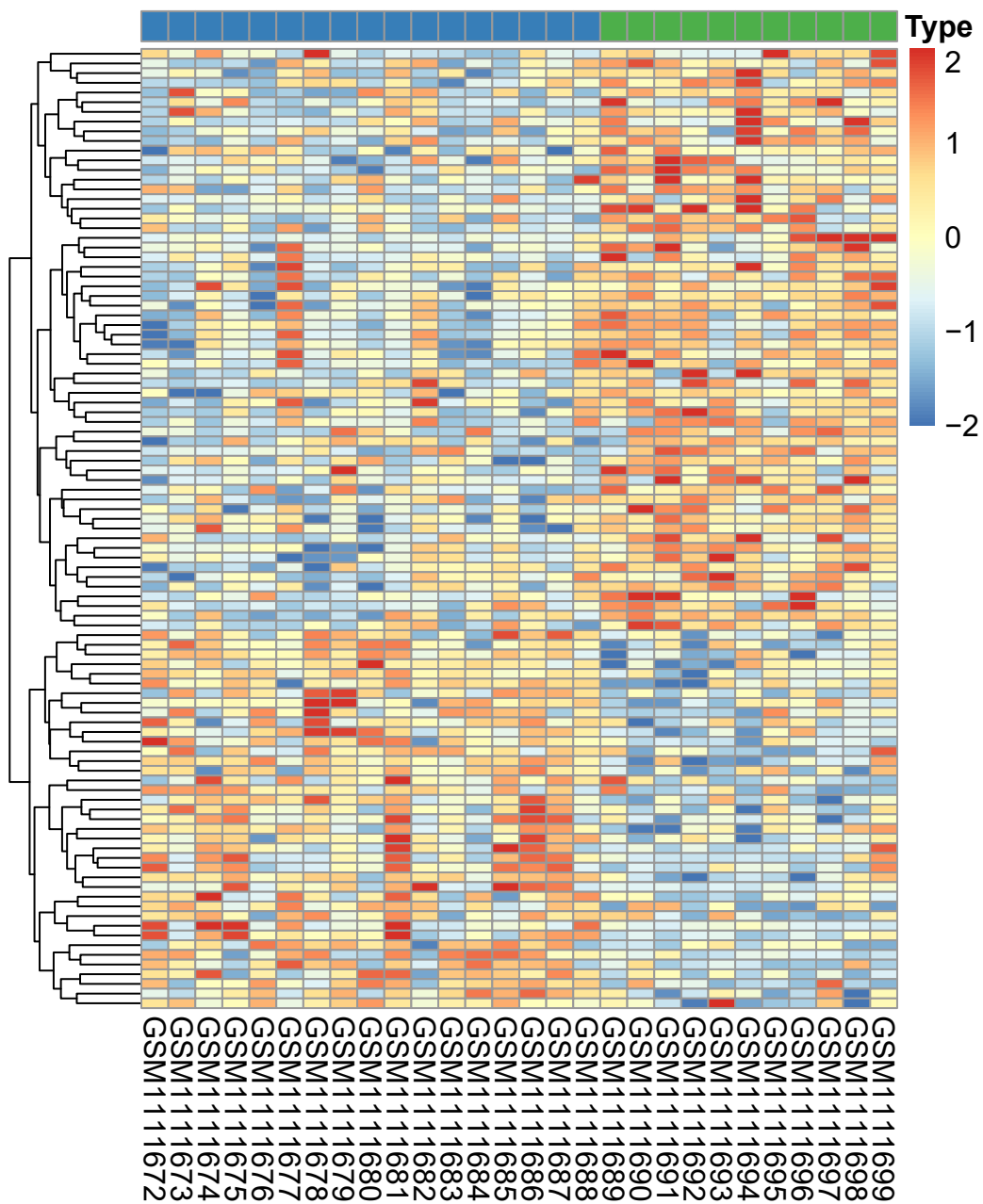

B

hub miRNA

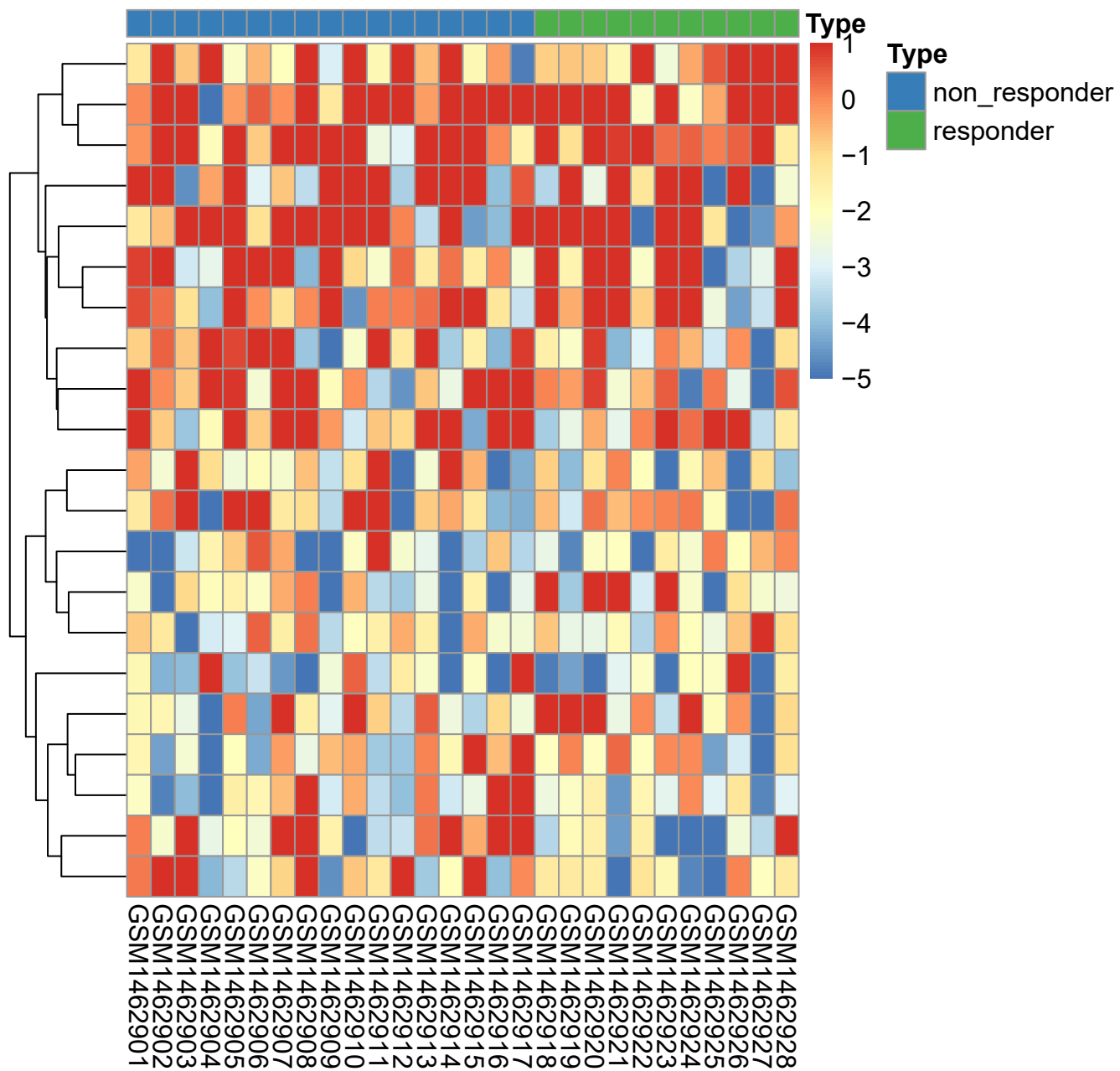

Supplement: Figure S4 — A. 99 genes in the regulation network in GSE45670. B. 21 miRNAs in regulation network in GSE59974. [file peerj-07-6668-s004.pdf]
