# Supplementary material for: Transcriptional factor regulation network and competitive endogenous RNA (ceRNA) network determining response of esophageal squamous cell carcinomas to neoadjuvant chemoradiotherapy
Source: PeerJ. 2019 Mar 29;7:e6668. doi: 10.7717/peerj.6668 (PMC6442670; doi:10.7717/peerj.6668)
Supplement: Table S1 [file peerj-07-6668-s005.docx]

| Gene.symbol | P.Value | logFC |
| --- | --- | --- |
| MRAP2 | 7.15E-06 | -3.5734842 |
| MMP13 | 0.01187482 | 2.8235502 |
| ZIC1 | 0.03386892 | -2.6486702 |
| CA9 | 0.00010091 | 2.5299555 |
| PRR9 | 0.00940504 | 2.4841526 |
| INHBA | 0.00989866 | 2.4678155 |
| LINC00942 | 0.02579747 | -2.4538267 |
| NKAIN2 | 0.00465696 | -2.4301048 |
| LOC101927870 | 0.00453948 | -2.4114438 |
| MMP10 | 0.00591266 | 2.3826068 |
| LCE3D | 0.00038451 | 2.3460391 |
| CDSN | 0.00121617 | 2.3149291 |
| AKR1C1 | 0.00193837 | -2.3010349 |
| ADH1B | 0.01364845 | -2.2832261 |
| MMP1 | 0.00703725 | 2.2760374 |
| SPRR2G | 0.00135206 | 2.2646306 |
| CTAG2 | 0.03006982 | 2.2417443 |
| WIF1 | 0.03473921 | -2.2165117 |
| SPOCK3 | 0.04619175 | -2.1687819 |
| CAPN13 | 0.0009637 | -2.0968735 |
| L1CAM | 0.00321632 | 2.0919518 |
| DMRT3 | 0.01055495 | -2.0877494 |
| JAKMIP3 | 0.03115529 | -2.0815826 |
| ERICH3 | 0.00241583 | 2.0750736 |
| FGFBP2 | 0.03812779 | -2.05437 |
| KRTAP5-AS1 | 0.00298683 | -2.044635 |
| TNNT1 | 0.00387623 | 2.0278826 |
| IGFL2 | 0.01723923 | 2.0269893 |
| AP3B2 | 0.00422996 | 2.0219855 |
| SERPINE1 | 0.01163454 | 2.0105791 |
| ANXA10 | 0.0170444 | -2.0101599 |
| UPK1B | 0.02653386 | -1.9750359 |
| LINC01553 | 0.00084206 | 1.9665786 |
| WDR49 | 0.02513933 | -1.956909 |
| ZFHX4-AS1 | 0.00108273 | 1.9566596 |
| CDH2 | 0.00637456 | 1.9539173 |
| RSPO2 | 0.0060247 | 1.938487 |
| CDH18 | 0.00836752 | 1.9311321 |
| ERVMER61-1 | 0.0031761 | 1.9286633 |
| ANGPTL3 | 5.15E-05 | 1.9081053 |
| TENM3 | 0.00018809 | 1.894325 |
| CYP26A1 | 0.02127098 | -1.877998 |
| APCDD1L-AS1 | 0.03441715 | 1.8667338 |
| USP2 | 0.00218061 | 1.8656216 |
| DGKB | 0.00026444 | -1.8648236 |
| CCDC8 | 0.00173744 | 1.8635212 |
| DISP2 | 0.0039069 | 1.8630866 |
| PHF21B | 0.01377177 | 1.8620522 |
| PPP4R4 | 0.01023962 | 1.8570971 |
| MAGEC2 | 0.01341832 | 1.8550116 |
| CYP4F3 | 0.00154559 | -1.8395773 |
| TDH | 0.0481479 | -1.8369348 |
| CNTNAP4 | 0.01077416 | 1.8331962 |
| SUGCT | 0.00739145 | 1.819288 |
| GART | 0.00724767 | 1.8179522 |
| ELAVL2 | 0.00810222 | 1.7987194 |
| DNAH5 | 0.01074324 | 1.7950201 |
| DSCAM | 0.0007839 | 1.7938036 |
| TLE2 | 0.00011534 | -1.7850412 |
| SOX2-OT | 0.006398 | -1.782518 |
| RPS6KA6 | 0.00703307 | -1.7647286 |
| QSOX2 | 2.89E-05 | 1.7570757 |
| LOC105377276 | 0.00441141 | 1.7479416 |
| GLCCI1 | 0.00035233 | 1.7454649 |
| SERPINI2 | 0.0144701 | -1.7449456 |
| SCN9A | 0.04403872 | -1.7375609 |
| RHEBL1 | 0.00066873 | 1.7350423 |
| WFDC12 | 0.00468699 | 1.7344032 |
| IGSF10 | 0.0005776 | -1.7336654 |
| ADAMTS9-AS2 | 0.00014241 | -1.7294794 |
| SCUBE2 | 0.00648608 | -1.7229608 |
| DDAH1 | 0.00027563 | 1.7181424 |
| EPHA10 | 0.00198685 | 1.7133053 |
| SKP2 | 0.00026038 | 1.7123101 |
| CHP2 | 0.00516022 | -1.7117928 |
| BAGE | 0.00168868 | 1.6932859 |
| HHLA2 | 0.00869819 | -1.6782677 |
| SLC51A | 0.00665987 | -1.6769005 |
| NAALAD2 | 0.01793939 | 1.675359 |
| ALB | 0.00216271 | 1.6712385 |
| MBNL1 | 0.01417058 | -1.6689656 |
| BMP4 | 0.01067221 | -1.6674639 |
| LINC00598 | 0.00201102 | 1.6656711 |
| PSG5 | 0.02434523 | 1.6638539 |
| KRT37 | 0.00114528 | 1.6634403 |
| MCHR2-AS1 | 0.00723985 | 1.6567279 |
| TBC1D22B | 0.00032296 | -1.6530646 |
| PLEC | 0.00142216 | 1.651755 |
| TUBB1 | 0.01832246 | -1.6515179 |
| TAPT1 | 0.00621707 | -1.6512483 |
| LOC101930370 | 0.00064773 | -1.6483966 |
| SCIN | 0.03123346 | -1.6465033 |
| TF | 0.04663661 | -1.6459471 |
| SLC26A3 | 0.00605522 | -1.6417325 |
| LINC00960 | 0.00301542 | -1.6404144 |
| MCF2L | 0.02193262 | -1.6332971 |
| GOLGA7B | 0.02984635 | 1.6330673 |
| GPR158 | 0.01860075 | 1.6304038 |
| CLDN8 | 0.00207396 | -1.6233162 |
| ABCA13 | 0.03369271 | -1.6178878 |
| FCHSD1 | 0.00211103 | 1.6134025 |
| C18orf21 | 0.00841723 | 1.6106219 |
| KRTDAP | 0.02236739 | 1.6093729 |
| COL12A1 | 0.00389185 | 1.6014818 |
| NGF | 0.00092862 | 1.5951279 |
| C1QTNF6 | 0.00016186 | 1.5899076 |
| PPARGC1A | 0.02994267 | -1.5861123 |
| LOC100996404 | 0.01110028 | -1.5819361 |
| PRR15 | 0.00938853 | -1.5814922 |
| LOC101929162 | 0.00585345 | 1.5794199 |
| VPS53 | 0.00902272 | -1.5773431 |
| LOC643659 | 0.00381154 | 1.5756901 |
| DAZL | 0.01636254 | 1.5751047 |
| CSRNP3 | 0.00948037 | -1.5700692 |
| SEZ6L2 | 0.02384826 | 1.5698193 |
| ZDHHC11 | 0.01144605 | -1.5630057 |
| EPHB2 | 0.00070559 | 1.5629947 |
| PLEKHS1 | 0.02755528 | 1.5614096 |
| TEX9 | 0.01885012 | 1.5590639 |
| KLK5 | 0.00063733 | 1.5589493 |
| KCNH8 | 0.02867992 | -1.5575674 |
| SOST | 0.03817236 | -1.5413203 |
| KLF12 | 0.00303833 | -1.5410275 |
| ANKRD7 | 0.00723264 | 1.540722 |
| EHD4 | 0.00480258 | 1.5391653 |
| ZNRD1ASP | 0.00306641 | -1.5383565 |
| IQGAP2 | 0.01501217 | 1.5376408 |
| EPYC | 0.00847767 | -1.537385 |
| PTHLH | 0.0281501 | 1.5364893 |
| KLF14 | 0.00184325 | 1.5337375 |
| CTNNA2 | 0.00586108 | 1.5332488 |
| CLMP | 0.00643389 | 1.5328927 |
| NTRK2 | 0.00596423 | -1.5306623 |
| LINC01021 | 0.03392366 | 1.5249039 |
| CAMK2B | 0.00644139 | -1.5245868 |
| IL32 | 0.00151174 | 1.5226161 |
| GPX2 | 0.02180577 | -1.5195336 |
| NRP1 | 0.01696465 | 1.5170084 |
| PAK5 | 0.03920655 | -1.5162234 |
| ZNF777 | 0.00019243 | 1.513481 |
| LOC101927292 | 0.01344217 | -1.5116192 |
| FLJ22763 | 0.02099617 | 1.5108358 |
| IL1RL1 | 0.00697571 | 1.5086515 |
| FAXC | 0.00056603 | -1.5075783 |
| RRBP1 | 0.00865754 | 1.5073515 |
| hsa-miR-873-3p | 0.0123641 | 9.50364 |
| hsa-miR-548l | 0.0024111 | 8.9868297 |
| hsa-miR-4464 | 0.0042012 | 8.7020439 |
| hsa-miR-890 | 0.0116099 | -8.6051527 |
| hsa-miR-593-5p | 0.0145514 | -8.4113971 |
| hsa-miR-548i | 0.0052399 | -8.3233267 |
| hsa-miR-491-5p | 0.0012368 | -7.9123948 |
| hsa-miR-3691-5p | 0.0196539 | 7.835409 |
| hsa-miR-4266 | 0.0038426 | 7.6818157 |
| hsa-miR-3675-5p | 0.0084494 | -7.6805932 |
| hsa-miR-644a | 0.0296275 | 7.5589028 |
| hsa-miR-92b-5p | 0.0220411 | 7.5403745 |
| hsa-miR-2682-5p | 0.0056332 | 6.6038484 |
| hsa-miR-554 | 0.0089928 | 6.571893 |
| hsa-miR-4727-5p | 0.0154541 | -6.5666677 |
| hsa-miR-4503 | 1.49E-05 | -6.4607613 |
| hsa-miR-1912 | 0.0118201 | -6.3690376 |
| hsa-miR-4752 | 0.0092129 | -6.3525453 |
| hsa-miR-616-3p | 0.0125133 | 6.2240455 |
| hsa-miR-3201 | 0.0109124 | -6.1010398 |
| hsa-miR-3690 | 0.0077117 | 6.0969154 |
| hsa-miR-3929 | 0.0297057 | 6.0775759 |
| hsa-miR-1251 | 0.0216972 | 6.0582331 |
| hsa-miR-219-1-3p | 0.0008269 | 6.0074803 |
| hsa-miR-552 | 0.0469567 | 5.9978809 |
| hsa-miR-5586-3p | 0.0029111 | 5.9770318 |
| hsa-miR-4445-5p | 0.0050912 | 5.9655543 |
| hsa-miR-1248 | 0.047606 | -5.8955704 |
| hsa-miR-374a-3p | 0.0173671 | -5.8752109 |
| hsa-miR-4650-3p | 0.0006026 | 5.6920495 |
| hsa-miR-4724-5p | 0.0068529 | -5.6798266 |
| hsa-miR-3153 | 0.0063295 | 5.6533541 |
| hsa-miR-3123 | 0.0235607 | 5.6225292 |
| hsa-miR-499b-5p | 0.033984 | -5.5978328 |
| hsa-miR-1269a | 0.034403 | 5.4533161 |
| hsa-miR-510 | 0.0002344 | -5.3054688 |
| hsa-miR-4802-3p | 0.0416596 | -5.2149353 |
| hsa-miR-5702 | 0.0006838 | -5.1491557 |
| hsa-miR-548h-3p | 0.001744 | 5.1303786 |
| hsa-miR-5091 | 0.0056659 | -5.1262986 |
| hsa-miR-5587-3p | 0.0245283 | 5.1181929 |
| hsa-miR-5693 | 0.0465332 | -5.0965857 |
| hsa-miR-4715-3p | 0.027284 | 5.0897737 |
| hsa-miR-3122 | 0.0019305 | -5.0169283 |
| hsa-miR-4794 | 0.0179059 | -4.9931682 |
| hsa-miR-615-5p | 0.0030365 | -4.9772104 |
| hsa-miR-4300 | 0.0204428 | 4.9596721 |
| hsa-miR-548at-5p | 0.0189088 | 4.9563717 |
| hsa-miR-590-3p | 0.003696 | -4.9054073 |
| hsa-miR-3166 | 0.0055718 | 4.8929186 |
| hsa-miR-4435 | 0.0272736 | 4.8703811 |
| hsa-miR-137 | 0.0131977 | 4.8479086 |
| hsa-miR-383 | 0.0128572 | -4.8356367 |
| hsa-miR-92a-1-5p | 0.0082791 | -4.819976 |
| hsa-miR-19b-1-5p | 0.0295776 | -4.7949334 |
| hsa-miR-20b-3p | 0.0229638 | 4.7913246 |
| hsa-miR-4711-3p | 0.0108165 | 4.7615014 |
| hsa-miR-548av-5p | 0.0186961 | 4.7454409 |
| hsa-miR-548h-5p | 0.0179396 | 4.7240461 |
| hsa-miR-4693-3p | 0.0125125 | -4.6241308 |
| hsa-miR-3148 | 0.0043976 | 4.6026809 |
| hsa-miR-488-3p | 0.0045351 | 4.5959583 |
| hsa-miR-4501 | 0.0461402 | -4.5725137 |
| hsa-miR-302a-5p | 0.0490594 | 4.521488 |
| hsa-miR-5680 | 0.0008104 | 4.5192953 |
| hsa-miR-561-3p | 0.0411417 | 4.4653161 |
| hsa-miR-526b-3p | 0.0237056 | 4.4026792 |
| hsa-miR-661 | 0.0493048 | -4.3924494 |
| hsa-miR-148a-5p | 0.0322584 | 4.3619777 |
| hsa-miR-548n | 0.0171083 | 4.3306634 |
| hsa-miR-1297 | 0.0372199 | -4.2550373 |
| hsa-miR-4781-3p | 0.0008893 | 4.2328234 |
| hsa-miR-670 | 0.0317299 | 4.2324092 |
| hsa-miR-4264 | 0.0278583 | 4.215887 |
| hsa-miR-589-3p | 0.0001757 | -4.2088275 |
| hsa-miR-647 | 0.0454673 | 4.1842309 |
| hsa-miR-4760-5p | 0.0448355 | 4.1653353 |
| hsa-miR-600 | 0.0044852 | -4.1434695 |
| hsa-miR-4520b-3p | 0.0166097 | 4.1304222 |
| hsa-miR-3178 | 0.0151718 | -4.1300061 |
| hsa-miR-608 | 0.0259257 | -4.1249269 |
| hsa-miR-4424 | 0.0171045 | -4.123494 |
| hsa-miR-4474-5p | 0.0322403 | 4.1097866 |
| hsa-miR-548am-3p | 0.0469454 | -4.0752599 |
| hsa-miR-891a | 0.0167068 | -4.0565624 |
| hsa-miR-4708-5p | 0.0362786 | -4.0556937 |
| hsa-miR-4783-5p | 0.0103783 | -4.0508998 |
| hsa-miR-642a-5p | 0.0198133 | -3.9773339 |
| hsa-miR-378c | 0.00431 | 3.9129918 |
| hsa-miR-668 | 0.0271129 | -3.9009573 |
| hsa-miR-3157-5p | 0.0202429 | 3.889157 |
| hsa-miR-4671-3p | 0.0104453 | 3.8659046 |
| hsa-miR-4491 | 0.046603 | 3.8622931 |
| hsa-miR-4638-3p | 0.0163938 | 3.8464916 |
| hsa-miR-4256 | 0.0125598 | -3.7607583 |
| hsa-miR-5571-3p | 0.0158174 | -3.7568116 |
| hsa-miR-4777-3p | 0.0332395 | -3.7507999 |
| hsa-miR-4305 | 0.0005891 | -3.746399 |
| hsa-miR-1294 | 0.0036583 | 3.6980113 |
| hsa-miR-412 | 0.0085837 | -3.6727609 |
| hsa-miR-4799-3p | 0.0487374 | -3.664359 |
| hsa-miR-548av-3p | 0.002855 | 3.6483742 |
| hsa-miR-1293 | 0.0317943 | 3.5949302 |
| hsa-miR-4803 | 0.0005722 | 3.584966 |
| hsa-miR-3177-5p | 0.0038855 | 3.5680382 |
| hsa-miR-4725-3p | 0.0016298 | -3.539313 |
| hsa-miR-4420 | 0.0457946 | 3.5226716 |
| hsa-miR-372 | 0.0314762 | -3.4982521 |
| hsa-miR-597 | 0.023601 | 3.4928576 |
| hsa-miR-548b-3p | 0.0010408 | 3.4689398 |
| hsa-miR-4751 | 0.0289074 | 3.4642334 |
| hsa-miR-4790-3p | 0.0335802 | 3.4174962 |
| hsa-miR-451b | 0.0261441 | 3.3914051 |
| hsa-miR-571 | 0.0301919 | -3.3852711 |
| hsa-miR-4705 | 0.0018212 | -3.3816953 |
| hsa-miR-1277-3p | 0.0075422 | 3.3377664 |
| hsa-miR-3184-5p | 0.0001237 | -3.2758525 |
| hsa-miR-4666a-3p | 0.0359411 | -3.2726965 |
| hsa-miR-1250 | 0.0074336 | 3.2695209 |
| hsa-miR-1247-5p | 0.0288615 | 3.2525889 |
| hsa-miR-3919 | 0.0230809 | 3.2464103 |
| hsa-miR-4302 | 0.0051868 | -3.2384747 |
| hsa-miR-4761-5p | 0.0190611 | 3.2350695 |
| hsa-miR-3617-5p | 0.0154269 | 3.2314657 |
| hsa-miR-3618 | 0.0085474 | 3.2304102 |
| hsa-miR-1228-5p | 0.0292598 | 3.2158812 |
| hsa-miR-5191 | 0.0099259 | -3.214134 |
| hsa-miR-4529-3p | 0.0272794 | 3.2021486 |
| hsa-miR-2278 | 0.0179913 | 3.1992083 |
| hsa-miR-5192 | 0.0356957 | 3.1430547 |
| hsa-miR-588 | 0.0121397 | -3.1363442 |
| hsa-miR-5687 | 0.0337152 | -3.1184023 |
| hsa-miR-4283 | 0.0269077 | 3.1097696 |
| hsa-miR-522-3p | 0.0034335 | 3.0986474 |
| hsa-miR-642b-5p | 0.0054576 | 3.0793656 |
| hsa-miR-1256 | 0.0070973 | -3.0761238 |
| hsa-miR-4684-5p | 0.0167525 | 3.0613795 |
| hsa-miR-5011-3p | 0.0078254 | 3.0252672 |
| hsa-miR-422a | 0.0110825 | 3.0202655 |
| hsa-miR-4713-5p | 0.0298712 | 3.0153133 |
| hsa-miR-593-3p | 0.0441789 | 3.0143958 |
| hsa-miR-1276 | 0.0002717 | 2.9979341 |
| hsa-miR-3616-5p | 0.0088138 | -2.9973065 |
| hsa-miR-802 | 0.0475964 | -2.990957 |
| hsa-miR-1258 | 0.0011261 | -2.9757936 |
| hsa-miR-5586-5p | 0.0455005 | 2.9591248 |
| hsa-miR-4329 | 0.0233975 | -2.9564278 |
| hsa-miR-302f | 0.0158211 | -2.9546213 |
| hsa-miR-3689a-5p | 0.009254 | 2.9527208 |
| hsa-miR-432-3p | 0.0098833 | -2.9422558 |
| hsa-miR-1292-5p | 0.0106088 | -2.9354977 |
| hsa-miR-124-5p | 0.0335522 | -2.902948 |
| hsa-miR-548s | 0.0309693 | -2.9005656 |
| hsa-miR-3943 | 0.0286992 | -2.8908085 |
| hsa-miR-561-5p | 0.023461 | -2.8672864 |
| hsa-miR-518d-3p | 0.0426942 | -2.8304981 |
| hsa-miR-3182 | 0.0012356 | 2.82725 |
| hsa-miR-206 | 0.0283064 | 2.8260895 |
| hsa-miR-595 | 0.0481535 | 2.8233645 |
| hsa-miR-93-3p | 0.0358517 | -2.80535 |
| hsa-miR-2115-3p | 0.0339295 | -2.802204 |
| hsa-miR-4715-5p | 0.0485162 | 2.8010478 |
| hsa-miR-2682-3p | 0.0035715 | -2.7881953 |
| hsa-miR-4512 | 0.0074802 | -2.7760611 |
| hsa-miR-607 | 0.017603 | -2.7620845 |
| hsa-miR-138-5p | 0.0160144 | -2.7229635 |
| hsa-miR-5585-5p | 0.0026826 | -2.7216041 |
| hsa-miR-3145-5p | 0.0184351 | -2.7062705 |
| hsa-miR-4686 | 0.0366847 | -2.6777528 |
| hsa-miR-4793-3p | 0.0115587 | 2.6556258 |
| hsa-miR-4493 | 0.0169977 | 2.6448522 |
| hsa-miR-3663-5p | 0.0117123 | 2.6417919 |
| hsa-miR-5187-5p | 0.012093 | 2.6071687 |
| hsa-miR-3124-5p | 0.0397522 | -2.5993791 |
| hsa-miR-924 | 0.0154774 | 2.5782446 |
| hsa-miR-195-3p | 0.0130518 | 2.5611132 |
| hsa-miR-4727-3p | 0.0106159 | 2.5484734 |
| hsa-miR-4694-5p | 0.0110426 | 2.5412562 |
| hsa-miR-519d | 0.001663 | -2.5328121 |
| hsa-miR-603 | 0.0391247 | 2.5202195 |
| hsa-miR-3064-3p | 0.0187439 | -2.5185466 |
| hsa-miR-1321 | 0.0120101 | 2.516716 |
| hsa-miR-1185-5p | 0.0109992 | -2.5117175 |
| hsa-miR-548aq-5p | 0.0141118 | 2.5092363 |
| hsa-miR-4315 | 0.0243981 | 2.5060149 |
| hsa-miR-4704-3p | 0.047092 | 2.5014795 |
| hsa-miR-1538 | 0.0477615 | 2.499879 |
| hsa-miR-4676-5p | 0.0349435 | 2.4826704 |
| hsa-miR-4638-5p | 0.020632 | -2.4822816 |
| hsa-miR-548d-5p | 0.0275093 | -2.4723484 |
| hsa-miR-4509 | 0.0459698 | -2.4695373 |
| hsa-miR-518e-5p | 0.0375298 | 2.4562394 |
| hsa-miR-101-5p | 0.0430034 | -2.4513497 |
| hsa-miR-5580-5p | 0.0389768 | -2.4494031 |
| hsa-miR-4664-5p | 0.0312439 | -2.4491361 |
| hsa-miR-520a-3p | 0.0052397 | -2.4378691 |
| hsa-miR-4536-3p | 0.0222039 | -2.4088527 |
| hsa-miR-4450 | 0.0381771 | 2.3983172 |
| hsa-miR-138-1-3p | 0.0320311 | -2.3977278 |
| hsa-miR-4252 | 0.0021526 | 2.3709081 |
| hsa-miR-1197 | 0.0431925 | 2.3691031 |
| hsa-miR-573 | 0.0301551 | 2.3545695 |
| hsa-miR-4458 | 0.009513 | 2.354266 |
| hsa-miR-656 | 0.0148816 | -2.3514393 |
| hsa-miR-5686 | 0.0462881 | -2.3481376 |
| hsa-miR-3189-3p | 0.0231491 | 2.330807 |
| hsa-miR-1252 | 0.0227157 | 2.329577 |
| hsa-miR-3677-3p | 0.0040549 | 2.2911241 |
| hsa-miR-371a-3p | 0.0160329 | 2.2478318 |
| hsa-miR-4639-5p | 0.0138966 | -2.2024922 |
| hsa-miR-517c-3p | 0.0493121 | 2.1799698 |
| hsa-miR-4280 | 0.0107321 | 2.1769264 |
| hsa-miR-548as-3p | 0.0219763 | -2.1695269 |
| hsa-miR-4659a-3p | 0.04395 | 2.114113 |
| hsa-miR-302c-3p | 0.0263933 | 2.1108798 |
| hsa-miR-633 | 0.0261086 | -2.0708848 |
| hsa-miR-4719 | 0.0104882 | -2.0460546 |
| hsa-miR-888-5p | 0.0332703 | 2.0404174 |
| hsa-miR-380-3p | 0.0217769 | -2.0267846 |
| hsa-miR-4446-3p | 0.0381815 | 2.0247035 |
| hsa-miR-521 | 0.032019 | 2.0224142 |
| hsa-miR-551b-3p | 0.0105336 | -2.0206539 |
| hsa-miR-4643 | 0.0180341 | -2.0204702 |
| hsa-miR-122-3p | 0.0460428 | -2.0172705 |
| hsa-miR-570-3p | 0.0124836 | -2.0057035 |
| hsa-miR-192-3p | 0.0306881 | -1.9771746 |
| hsa-miR-1273d | 0.0180911 | 1.9732575 |
| hsa-miR-4524b-5p | 0.028613 | -1.9614824 |
| hsa-miR-1224-3p | 0.0414406 | -1.9453873 |
| hsa-miR-4736 | 0.045713 | 1.9360009 |
| hsa-miR-645 | 0.0387872 | 1.9335974 |
| hsa-miR-654-5p | 0.0350513 | -1.8863566 |
| hsa-miR-4660 | 0.0129618 | 1.8716159 |
| hsa-miR-5090 | 0.0201897 | 1.8570201 |
| hsa-miR-548u | 0.0415195 | -1.8512975 |
| hsa-miR-4304 | 0.0389672 | 1.840597 |
| hsa-miR-4421 | 0.0483097 | 1.8167064 |
| hsa-miR-3155b | 0.0290093 | 1.7887274 |
| hsa-miR-1255b-5p | 0.0292127 | 1.7790848 |
| hsa-miR-5572 | 0.0409047 | 1.7632486 |
| hsa-miR-4785 | 0.0223514 | 1.7386689 |
| hsa-miR-5584-5p | 0.0245735 | 1.7173367 |
| hsa-miR-370 | 0.0183394 | 1.7032884 |
| hsa-miR-191-5p | 0.0238697 | -1.7030039 |
| hsa-miR-3622b-5p | 0.0219301 | 1.6789496 |
| hsa-miR-548x-3p | 0.0047067 | -1.6265545 |
| hsa-miR-204-3p | 0.0136177 | 1.616368 |
